# Operational research for strengthening leadership and advancing universal health coverage in India

**DOI:** 10.5588/pha.26.0048

**Published:** 2026-08-24

**Authors:** J.P. Tripathy, P.P. Joshi, M. Vijayageetha, P.R. Deshmukh, K. Niraimathi, S. Satyanarayana, A. Groover, A. Alwani, K.C. Prajitha, A.M.V. Kumar, A. Seshachalam, P. Chinnakali, P. Naik, P. Thekkur, S. Cyriac, A.D Harries, C.M. Halleux, R. Zachariah, M. Lacerda

**Affiliations:** 1 All India Institute of Medical Sciences, Nagpur, India;; 2 Evidentia Research Solutions, Chennai, India;; 3 ICMR-National Institute for Research in Tuberculosis (ICMR-NIRT), Department of Health Research, Ministry of Health and Family Welfare, Government of India, Chennai, India;; 4 Division of Implementation Research, Extramural Division, Indian Council of Medical Research, New Delhi, India;; 5 The Union South-East Asia Office, New Delhi, India;; 6 International Union against Tuberculosis and Lung Disease (The Union), Paris, France;; 7 Yenepoya Medical College, Yenepoya (Deemed to be University), Mangaluru, India;; 8 Dr GVN Cancer Institute, Trichy, India;; 9 Department of Preventive and Social Medicine, JIPMER Pondicherry, India;; 10 Department of Medical Oncology, Rajagiri Hospital, Kochi, India;; 11 London, School of Hygiene and Tropical Medicine, London, UK;; 12 UNICEF/UNDP/World Bank/WHO Special Programme for Research and Training in Tropical Diseases (TDR), WHO, Geneva, Switzerland.

**Keywords:** SORT IT, tuberculosis, cancer screening, diabetes, anemia, transplant care, leprosy, PTLD, lymphatic filariasis

## Abstract

This issue of *Public Health Action* includes a series of articles on ‘Research leadership in India’, a Structured Operational Research and Training Initiative (SORT IT), led by TDR, as a model for strengthening operational research capacity within health systems globally. Conducted in 19 institutions in India, it transforms frontline health challenges into research that informs policy and practice. The 11 articles in this issue address public health challenges while enhancing self-reliance and people-centered health approaches as drivers for universal health coverage and equity. By placing individuals, communities and frontline realities at the center of research, the series reaffirms that meaningful and lasting health system transformation begins with listening, learning, and leading from within.

## Graphical abstract

**Figure:**
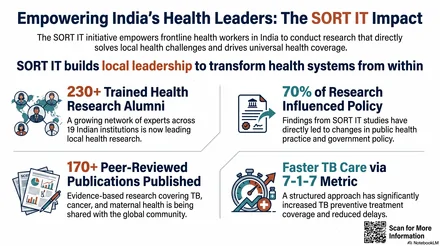

The Structured Operational Research and Training Initiative (SORT IT), led by the Special Programme for Research and Training in Tropical Diseases (TDR), exemplifies how global partnerships can build sustainable capacity in operational research.^1^ At its core, since 2012, SORT IT seeks to equip health systems with the knowledge, skills, and leadership required to improve the delivery, quality, and equity of public health services.^2^ This issue of *Public Health Action* includes a series of articles on ‘Research leadership in India’, a compelling body of work that demonstrates how operational research strengthens health systems and accelerates progress toward universal health coverage.^3^ Central to this effort is a collaborative model that brings together frontline health workers, non-governmental organizations, academic institutions, and postgraduate trainees in India.

What distinguishes this series is that it is grounded in real-world challenges. The research questions that shaped these studies emerged from constraints faced by frontline health workers and decision-makers across India. By transforming such everyday barriers into research opportunities, these efforts not only generate evidence but also cultivate problem-solving capacity where it is most needed.^4–14^ Collectively, the studies span several linked themes: infectious diseases and long-term outcomes (TB survivorship,^13^ TB among people with diabetes,^9^ and delayed leprosy detection^11^); health service delivery (integration of cervical cancer screening in STI clinics,^6^ ambulance responsiveness,^4^ and safety of autologous transplant care^8^); access and supportive care (nutritional and social support for TB,^7^ barriers to parenteral therapy for maternal anemia,^10^ and access to affordable generics^5^); disability prevention (lymphatic filariasis^14^); and disease burden (patterns of cancer mortality^12^).

Hosted by the All India Institute of Medical Sciences (AIIMS), Nagpur, this national SORT IT brought together 19 institutions in a collaborative partnership to boost operational research. Key partners included The Indian Council of Medical Research, The International Union Against Tuberculosis and Lung Disease, Jawaharlal Institute of Postgraduate Medical Education & Research, and several leading clinical and research institutions across the country. Together, they created a vibrant learning ecosystem that links research with policy and practice. In line with the World Health Report of the WHO,^3^ this SORT IT is guided by a clear and compelling vision:

‘Research capacity and leadership should be built close to the supply and demand of health services so that it shapes health systems and improves policies and practices for greater public health impact.’ Adapted from the World Health Report^3^

The choice of Nagpur as the setting for this initiative adds a deeper symbolic resonance. Located near Sevagram Ashram, once the headquarters of visionary leaders such as Mahatma Gandhi and Jamnalal Bajaj, the region carries a legacy of service, simplicity and transformative social change. Sevagram was not merely a place; it was a living laboratory where ideas of self-reliance and people-centered development were translated into action. Additionally, this is in line with the current government’s stimulus for developing ‘Atmanirbhar Bharat Abhiyan’ (Self-reliant India).^15^

India now stands at a pivotal moment, well positioned to scale up the legacy of the SORT IT model within its health system. With a network of 236 trained alumni, many now leading institutions and research units across the country, there is a foundation for sustained, locally driven research capacity.^16,17^ The outputs speak for themselves: over 170 peer-reviewed publications have emerged from SORT IT trainees in India, and impact evaluation surveys indicate that nearly 70% have influenced policy and practice.^16^ With this in mind, each article in this series includes a short video abstract and explanatory infographic. These elements are intended to allow researchers, policymakers and readers lacking subject-matter expertise to explore and quickly understand each article. Our hope is that this will lead to even greater engagement and promote adoption into policy and practice.

Beyond these outputs, the SORT IT model has also empowered individuals, with approximately half of all trainees reporting that they continued to conduct independent research, while 40% have stepped into mentorship roles in new SORT IT courses, nurturing the next generation of researchers.^16,17^ The Nagpur initiative also offers an illustration of the evolution of mentors. Studies were led or co-led by former SORT IT trainees who provided senior leadership and mentorship roles, demonstrating not only continuity but maturing leadership capacity. These are indicators of capacity built, not just in terms of numbers, but in confidence, ownership, and sustained engagement. A compelling example of operational research with associated patient and societal impact is the use of the ‘7-1-7’ timeliness metric among household contacts of TB patients in India: identifying household contacts within 7 days; screening them within 1 day; and initiating TB preventive treatment (TPT) within 7 days.^18^ This structured approach is associated with a significant increase in TPT coverage and reduced delays in initiation, contributing to TB prevention in this high-risk group.^19^ It also informed global policy, with the WHO recommending adoption of these timeliness metrics to ensure rapid contact management and timely TPT uptake worldwide.^20^

Looking ahead, the priority is to consolidate these gains while reaffirming the principles that have underpinned SORT IT’s success. 1) Research priorities must remain locally defined, anchored in the ethos of local research, local ownership, and local solutions. 2) Stronger synergies between academic institutions and public health programmes should be fostered by pairing professionals from both sectors within each project, enabling a convergence of methodological rigor and practical insight. 3) Partnerships should be expanded further to strengthen training-of trainers and community engagement while also bringing in the private sector and donor communities. 4) Finally, successive cycles of SORT IT should effectively communicate research findings to policy makers and assess the downstream impact of research, thereby advancing the concept of “operational research on operational research”.^21^

## CONCLUSION

This initiative embodies a broader and enduring vision: the pursuit of self-reliance and people-centered health approaches as essential drivers to accelerate efforts towards achieving universal health coverage and equity. By placing individuals, communities and frontline realities at the center of research, it reaffirms a fundamental principle: that meaningful and lasting transformation in health systems must begin with listening, learning, and leading from within.

## References

[1] TDR . SORT IT operational research and training. https://tdr.who.int/activities/sort-it-operational-research-and-training

[2] Zachariah R , Het al . Operational research in low-income countries: what, why, and how? Lancet Infect Dis 2009; 9(11): 711-717. 10.1016/S1473-3099(09)70229-4 19850229

[3] World Health Organization . The World Health Report 2013: Research for Universal Health Coverage. Geneva: WHO, 2013.

[4] Mirza M. , The prehospital pulse: high responsiveness, deployment, and utilization of ambulance services in India. Public Health Action 2026;16(3):188-194.

[5] Sathiyamoorthy R. , Gaps in access and trust: essential medicine availability, affordability, and barriers to generic medicine use. Public Health Action 2026;16(3):195-201.

[6] Yadav P. , Leveraging sexually transmitted infection clinics for cervical cancer screening: evidence from India. Public Health Action 2026;16(3):202-208.

[7] Chavda P. , Food support for TB care: coverage, outcomes and implementation lessons from the Nikshay Mitra scheme in India. Public Health Action 2026;16(3):209-215.

[8] Joy A. , Multimodal melphalan delivery: improved safety and outcomes with autologous myeloma transplant in India. Public Health Action 2026;16(3):216-221.

[9] Devaraj L. , Dynamic TB infection pattern: high conversion and reversion among persons with diabetes in India. Public Health Action 2026;16(3):222-228.

[10] Ramaswamy G. , Enhancing parenteral therapy for anemia among pregnant women in rural India. Public Health Action 2026;16(3):237-243.

[11] Das S. , Understanding patient pathways and care delays in India: implications for early leprosy detection. Public Health Action 2026;16(3):237-243.

[12] Doshi A. , Why do cancer patients die? A decade of experience on trends, profile, causes and timing of deaths in India. Public Health Action 2026;16(3):244-249.

[13] Subramanian S. , Cured but not healed: high burden of pulmonary disability and multimorbidity among TB survivors in India. Public Health Action 2026;16(3):250-256.

[14] Bashar A. , Assessing morbidity management and disability prevention for lymphatic filariasis in India. Public Health Action 2026;16(3):257-264.

[15] Ministry of Tribal Affairs - Government of India . Aatmanirbhar Bharat Abhiyan. https://tribal.nic.in/atmanirbhar-bharat.aspx

[16] Zachariah R , Implementation research for strengthening health systems in India. Indian J. Med Res 2024; 159(3&4): 308-313. 10.25259/IJMR_260_24 PMC11414784 39361804

[17] Seshachalam A , SORT IT empowers oncology clinicians to boost research capacity and advance universal health coverage in India. Public Health Action 2025;15(4):183-185. 10.5588/pha.25.0039 PMC12687122 41376677

[18] Nair D , Implementing timeliness metrics for household contact tracing and TB preventive treatment through TB champions in the public sector, India: an explanatory mixed-methods study. BMJ Open;15(11):e097935. 10.1136/bmjopen-2024-097935 PMC12588006 41248341

[19] Harries AD , t TB preventive therapy: uptake and time to initiation during implementation of '7-1-7' IJTLD Open 2024;1(4):189-191. 10.5588/ijtldopen.24.0101 PMC11231823 38988406

[20] World Health Organization . WHO operational handbook on tuberculosis: module 1: prevention: tuberculosis preventive treatment, 2nd ed. Geneva, WHO, 2024. 39298638

[21] Zachariah R , Operational Research on Operational Research: Assessing the Impact of the Structured Operational Research and Training Initiative on Tackling Antimicrobial Resistance in Ghana. Trop Med Infect Dis 2025;10(11):312. 10.3390/tropicalmed10110312 PMC12656594 41295577

